# Modulation of Brain Electroencephalography Oscillations by Electroacupuncture in a Rat Model of Postincisional Pain

**DOI:** 10.1155/2013/160357

**Published:** 2013-04-28

**Authors:** Jing Wang, Jing Wang, Xuezhu Li, Duan Li, Xiao-Li Li, Ji-Sheng Han, You Wan

**Affiliations:** ^1^Neuroscience Research Institute, Peking University, Beijing 100191, China; ^2^State Key Lab of Cognitive Neuroscience and Learning, Beijing Normal University, Beijing 100875, China; ^3^Institute of Electrical Engineering, Yanshan University, Qinhuangdao 066004, China; ^4^Key Laboratory for Neuroscience, Ministry of Education/Ministry of Health, Beijing 100191, China

## Abstract

The present study aimed to investigate how ongoing brain rhythmical oscillations changed during the postoperative pain and whether electroacupuncture (EA) regulated these brain oscillations when it relieved pain. We established a postincisional pain model of rats with plantar incision to mimic the clinical pathological pain state, tested the analgesic effects of EA, and recorded electroencephalography (EEG) activities before and after the EA application. By analysis of power spectrum and bicoherence of EEG, we found that in rats with postincisional pain, ongoing activities at the delta-frequency band decreased, while activities at theta-, alpha-, and beta-frequency bands increased. EA treatment on these postincisional pain rats decreased the power at high-frequency bands especially at the beta-frequency band and reversed the enhancement of the cross-frequency coupling strength between the beta band and low-frequency bands. After searching for the PubMed, our study is the first time to describe that brain oscillations are correlated with the processing of spontaneous pain information in postincisional pain model of rats, and EA could regulate these brain rhythmical frequency oscillations, including the power and cross-frequency couplings.

## 1. Introduction

Brain rhythmical oscillations in the low (delta, theta, and alpha) and high (beta and gamma) frequencies of electroencephalography (EEG) have been demonstrated to be linked to broad varieties of perceptual, sensorimotor, and cognitive operations [1]. Interaction of oscillations at different frequencies, for example, cross-frequency phase synchronization between alpha, beta, and gamma oscillations, could be observed during working memory, perception, and consciousness [2].

Pain, as a perception, is subserved by an extended network of brain areas [3], or different brain networks are involved in the perception of pain [4]. Previous studies on acute pain disclosed that painful stimulation altered the activities of different frequency oscillations [5, 6], including their power and phase couplings.

It is also noticed that most EEG neurophysiological studies on acute pain were based on phasic pain models with experimental pain induced by short-lasting, noninvasive painful stimuli (e.g., laser noxious heat stimulation). When we consider clinical situations, it is obvious that tonic pathological pain models could better mimic clinical pain than phasic pain models [7].

Postoperative incisional pain is common in clinic. In the rat model of incisional pain, persistent pain existed for several days after the hind paw incision with peripheral and central sensitization [8, 9]. On human subjects after the incision, an imaging study observed increased brain activities in the anterior cingulate cortex (ACC), the insular cortex, the thalamus, the frontal cortex, and the somatosensory cortex [10], and it would be useful to know how the brain EEG oscillation changes in tonic pathological pain states like postoperative pain (postincisional pain in the rat model).

Acupuncture has been widely used in clinical settings. Acupuncture or electroacupuncture (EA) has therapeutic effects in various painful conditions, and these effects could last for a long period of time even hours after acupuncture application being terminated [11–13]. Neuroimaging studies revealed that acupuncture or EA application elicited widespread changes in cerebrocerebellar brain regions [14, 15], largely overlapped with the neural networks for both pain transmission and perception. Acupuncture could directly affect EEG activities on healthy volunteers as well as on animals [16, 17]. Experimental and clinical evidence indicated that pain could affect cognitive processes [18], default-mode network dynamics [19] and even decreased the grey matter volume of brain regions [20]. To examine the neural consequences of acupuncture or EA treatment on pain, it would be useful to determine how acupuncture or EA treatment modulates cortical activities under tonic pathological pain conditions.

We established a rat model of plantar incision to mimic the clinic pain and observed the analgesic effects of EA on this model. On this basis, with EEG study, we further investigated changes of spontaneous brain oscillations in the incisional pain and the EA modulation on EEG oscillations.

## 2. Materials and Methods

All experimental procedures were in accordance with the guideline of the International Association for the Study of Pain [21] and were approved by the Animal Care and Use Committee of our university. The behavioral experimenters were kept blind.

### 2.1. Animals and Housing

Adult Sprague-Dawley male rats were provided by the Department of Experimental Animal Sciences of our university (30 rats for the behavior test, weighing 180–230 g; and 16 rats for EEG recordings, weighing 300–350 g at the beginning of the experiment). They were housed individually in cage with free access to food and water. The temperature was maintained at about 22°C under natural light/dark cycles. Rats were habituated to the environment and handled daily for one week before the experiment.

### 2.2. The Plantar Incisional Pain Model of Rats

The rat model of incisional pain employed a 1 cm longitudinal incision with muscle involvement [22]. Briefly, the animal was placed in a sealed glass container with 5% isoflurane mixed with air to induce anesthesia and delivered 1.5–2% isoflurane via a nose cone to maintain the anesthesia during the following surgical operation. The left hind paw of the rat was sterilized with 10% povidone-iodine, and a sterile no. 11 scalpel blade was used to make a 1 cm long incision through the skin and fascia of the plantar hind paw including the underlying muscles, beginning 0.5 cm from the heel. The wound was closed with two mattress sutures of 5–0 nylon, covering with the antibiotic ointment.

### 2.3. EA Application

Before the incisional surgery, the rat was loosely immobilized by the bandage on a metal mesh floor with the head, hind legs, and the tail protruding and habituated for 3-4 days, at least 20 min a day, in order to minimize the discomfort and the tension during the EA operation and application.

Two hours after the plantar incision, rats were divided into three groups: the restriction (incision) group, which received plantar incision in the left hind paw and was loosely immobilized by the bandage; the sham EA group, which received needling at “acupoints” without electrical stimulation after the incision; and the EA group, which received EA application bilaterally after the incision. EA was applied according to the routine procedure as in our previous reports [23–26]. Stainless-steel needles (0.4 mm in diameter, 4 mm in length) were inserted into the “acupoints” on each hind leg. Two commonly used acupoints, “Zusanli” (ST36, 4 mm lateral to the anterior tubercle of the tibia, which is marked by a notch) and “Sanyinjiao” (SP6, 3 mm proximal to the medial malleolus, at the posterior border of the tibia), were stimulated with square waves of 0.2 ms in pulse width and 2 Hz in frequency from a Han's Acupoint Nerve Stimulator (HANS, LH series, manufactured in our university). The EA intensities were increased in a stepwise manner at 1-2-3 mA, with each intensity lasting for 10 min.

### 2.4. Assessment of Mechanical Allodynia

Mechanical allodynia was assessed by measuring the 50% paw withdrawal threshold (PWT) as described in our previous reports [26, 27]. The 50% PWT in response to a series of *von *Frey filaments (Semmes-Weinstein Monofilaments, North Coast Medial Inc., San Jose, CA, USA) was determined by the up-down method [28]. The rat was restricted on a metal mesh floor covered with an inverted clear plastic cage (18 × 8 × 8 cm) and allowed a 15–20 min period for habituation. Eight *von *Frey filaments with approximately equal logarithmic incremental (0.224) bending forces were chosen (0.41, 0.70, 1.20, 2.00, 3.63, 5.50, 8.50, and 15.10 g). Each trial started with a *von *Frey force of 2.00 g delivered perpendicularly to the plantar surface of the left hind paw adjacent to the wound near the medial heel. An abrupt withdrawal of the foot during the stimulation or immediately after the removal of the filament was recorded as a positive response. Once a positive or a negative response was evoked, the next weaker or stronger filament was applied, respectively. This procedure was terminated until 6 stimuli after the first change in response occurred. The 50% PWT was calculated using the following formula:

(1) $$\begin{array}{c} 50\%\;\;\text{PWT}=\frac{10^{\left(X_{f}+k\delta \right)}}{10^{4}}, \end{array}$$

where *X*_*f*_ is the value of the last *von *Frey filament used (in log unit), *k* is a value measured from the pattern of positive/negative responses, and *δ* = 0.224 which is the average interval (in log unit) between the *von *Frey filaments [29]. The mechanical allodynia of the left hind paw was tested at 6 time points, that is, before the incision, after the incision but before the EA application, 10, 20, and 30 min during the EA application, and 30 min after the EA application. The data were analyzed with one-way ANOVA followed by Tukey post hoc test, and *P* < 0.05 was chosen as statistically significant level.

### 2.5. Electrode Implantation for EEG Recording

Sixteen male rats were anesthetized with sodium pentobarbital (50 mg/kg, *i.p.*). After removing the scalp of the rat and exposing the skull, 14 stainless steel screws (tip diameter 1 mm, impedance 300–350 Ω) with sockets were implanted bilaterally as epidural electrodes into the skull to record the cortical EEGs. The locations of these electrodes were determined by the method from Shaw et al. [30]: anterior frontal (FL1, FR1), anterior (A) +4.5 mm, lateral (L) ±1.5 mm; centrofrontal (FL2, FR2, PR1, PL1), A ±1.5 mm, L ±4.5 mm; lateral frontal (PL2, PR2), A 0.0 mm, L ±4.5 mm; frontooccipital (LFL, RFR, LPL, RPR), A −4.5 mm, L ±1.5 mm for LFL, RFR, and A −3.0 mm, L ±4.5 mm for LPL, RPR. The reference and the ground electrodes were positioned 2 mm and 4 mm caudal to the lambda, respectively (Figure S1 of the Supplementary Material available at http://dx.doi.org/10.1155/2013/160357). The electrodes were fixed to the skull with dental cement and had no any connections with muscles. Penicillin (6 × 10^4^ U, i.m.) was administrated for 3 consecutive days to prevent possible infection.

### 2.6. EEG Signals Collection and Analysis

After one week recovery from the surgery, rats were habituated to the restriction for 3-4 days as the above mentioned, then the EEG rats were randomly divided into EA group or control group (restriction only) (*n* = 8 for each group). Rats were loosely immobilized on a metal mesh floor for the convenience of EA application during EEG recordings. EEG signals were collected in awake rats during three sessions, that is, before the plantar incision, 1.5–2 h after the plantar incision, and after the EA application. Each session lasted for 25–30 min.

The ASA-Lab EEG/ERP recording system (ANT Inc., The Netherlands) was used. Data were analyzed offline with Matlab (The Mathworks, Natick, MA, USA) EEGLAB software. The movement artifact or the baseline drift was removed from all channels, and the channels with impedance values above 25 k Ohms were also discarded. The EEG signals were digitized at a sampling rate of 256 Hz, rereferenced to an average of residual channels, and filtered through a 1–45 Hz band pass to avoid the interference of 50 Hz signals.

The wavelet power spectrum was used to obtain the power of the on going EEG activities [31]. The Morlet wavelet transform was employed with the wavelet central angle frequency of 6 (*ω* = 6). Five spectral bands were examined: 1–4, 4–8, 8–13, 13–30, 30–45 Hz, corresponding to delta, theta, alpha, beta, and gamma bands, respectively [32, 33], with a step of 0.5 Hz.

The bispectral analysis, including the amplitude and the phase information, is used to quantify the degree of quadratic phase coupling (QPC) among different frequency components of a signal [34]. Bicoherence method is the normalized form of the bispectral analysis; it is independent of the amplitude of the signal, therefore; it can be used as an indicator of phase coupling in nonlinear signals. In this study, general harmonic wavelet bicoherence was employed to measure the comodulation of oscillations between two frequency bands [35]. Signals were divided into a series of 2-second epochs, with an overlap of 75%. For each epoch, bicoherence values were computed in all pairs of frequencies from 1 to 45 Hz, with a step of 1 Hz and a bandwidth of 2 Hz. The same epoch as the power analysis in the above was used to calculate the filtered wavelet bicoherence value (FIWBIC).

### 2.7. Statistical Analysis of EEG Data

For power spectral data, a paired *t*-test was used to analyze at which frequency ranges the change of the EEG power was significant. In the EA and the restriction groups, the change of power was expressed as the percentage relative to the power value during the session before the incision, which was defined as (After − Before)/Before × 100%, and paired *t*-tests were used in the interior-group. Unpaired *t*-tests were further used between the two groups.

For the wavelet bicoherence data, the statistical analysis was focused on their characteristics to determine if a significant difference existed between different recording sessions; so prior to the comparison, the total bicoherence value at the frequency bands (*f*_*j*_^*L*^ ≤ *f*_*j*_ ≤ *f*_*j*_^*U*^  and  *f*_*k*_^*L*^ ≤ *f*_*j*_ ≤ *f*_*k*_^*U*^) was extracted, which was defined as

(2) $$\begin{array}{c} b=\sum \sum b_{xxx}^{2}\left(f_{j},f_{k}\right), \end{array}$$

where *b*_*xxx*_ is the bicoherence value (FIWBIC). This value is a measure of the degree of QPC between frequency bands and can be used to measure the phase coupling strength between different waves [35]. Then, a Wilcoxon rank-sum test was conducted to determine significant difference, with *P* value less than 0.05 as a statistically significant standard.

## 3. Results

### 3.1. EA Treatment Attenuates Mechanical Allodynia in the Incisional Pain Model of Rats

**Figure 1:**
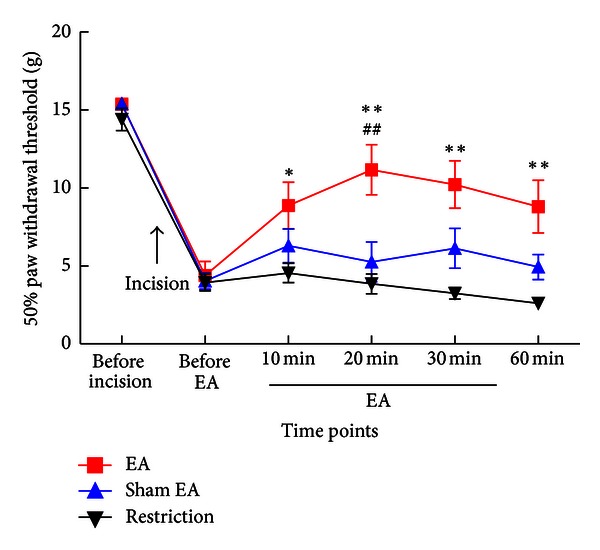
The effect of EA treatment on mechanical allodynia in the postincisional pain model of rats. Mechanical allodynia of the left hind paw was tested before incision, before EA treatment, 10, 20, and 30 min during EA, and 30 min after EA application. A 50% of paw withdrawal threshold (PWT) significantly increased after EA. *P < 0.05, **P < 0.01 compared with the restriction group; ^##^ *P* < 0.01 compared with the sham-treatment group. Data were presented as means ± SEM, *n* = 9-10.

We established the plantar incisional pain model of rats and applied 2 Hz EA to “Zusanli” and “Sanyinjiao” acupoints in bilateral sides of the hind paw. A 50% of PWT to calibrated *von* Frey filaments was measured over time. Mechanical allodynia was attenuated significantly during EA application, and this antinociceptive effect could last for at least 30 min after EA application compared to that in the sham EA group or in the restriction group (Figure 1).

### 3.2. Changes of EEG Oscillations in the Incisional Pain Model of Rats

**Figure 2:**
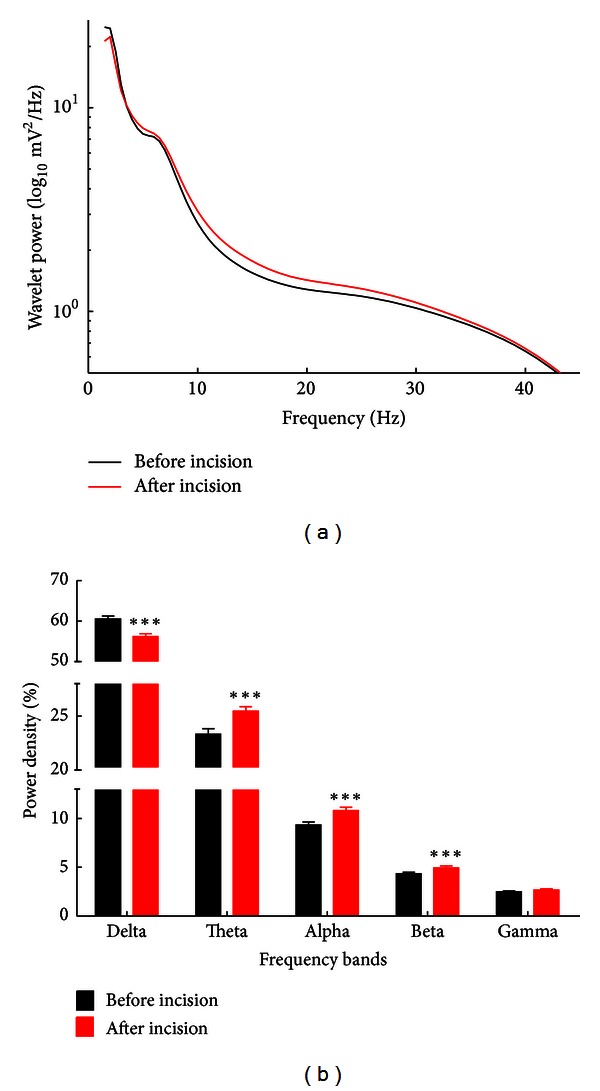
Changes of EEG power spectra in the postincisional pain model of rats. (a) The average of absolute EEG power before the incision (black line) and after the incision (red line). The *y*-axis represented power value, and *x*-axis represented frequency bands. (b) The relative power (normalized to the overall power) at 5 frequency bands. A significant increase of relative power at theta, alpha, and beta frequencies as well as a decrease at delta frequency was observed after the incision (red columns), compared with the power before the plantar surgery (black columns). **P < 0.01, ***P < 0.001 compared with before incision. All data were expressed as means ± SEM.

To examine the ongoing brain activities induced by the plantar incision, we calculated the power of different oscillations for each recording session (Figure 2). Compared with the session before the incision, the relative spontaneous EEG power of delta-frequency oscillation decreased, while the power in theta, alpha, and beta bands increased in rats after plantar incision. In addition, no significant change in gamma power was observed.

**Figure 3:**
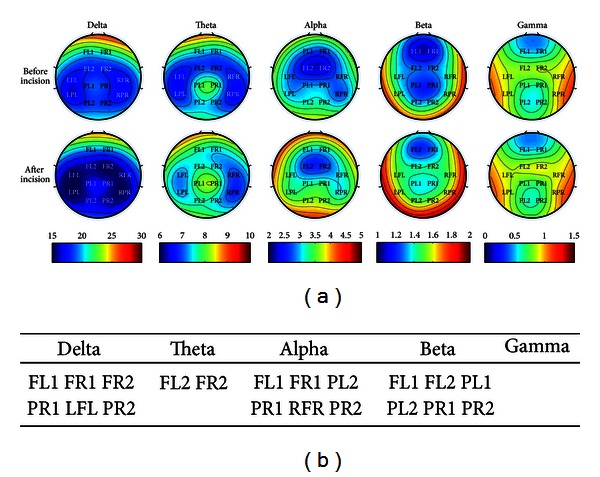
Locations of electrodes with significant EEG power change in the postincisional pain model of rats. (a) Topographic mapping of EEG during two sessions before (upper) and after (lower) the plantar incision. Averaged EEG power densities ranged from delta to gamma bands. Values were color-coded and plotted at the corresponding position on the planar projection of the epidural surface and interpolated between electrodes (dots). (b) Locations of electrodes showing statistically significant difference (*P* < 0.05) of power changes at different bands.

Furthermore, we compared the power of each channel at different frequency bands between different sessions. The corresponding topographies were shown in Figure 3(a). The locations where power changed significantly at different frequency bands during the sessions after the plantar incision and after EA application were summarized in Figure 3(b), respectively. We found that the changes mainly located at bilateral fronto-parietal lobes in low and medium frequency bands after the plantar incision.

**Figure 4:**
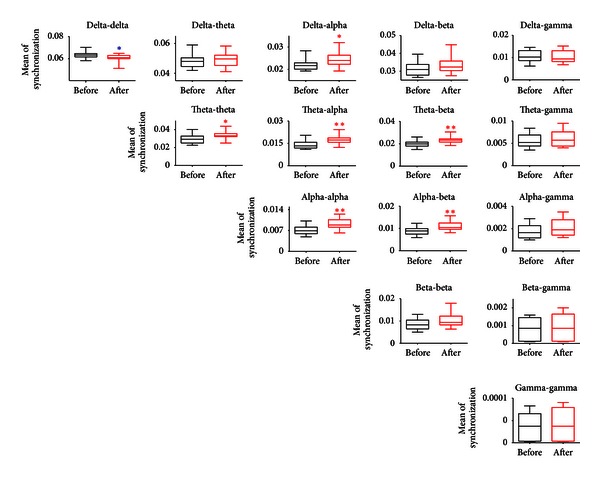
Phase couplings at different frequency bands: statistical analysis of the filtered wavelet bicoherence (FIWBIC) values in the postincisional pain model of rats. Boxplots stand for the phase coupling at local frequency bands, and the *y*-axis represents the mean of synchronization. The synchronization values increased between delta and alpha, between theta and theta/alpha/beta, between alpha and alpha/beta, whereas the value decreased at delta band after the plantar incision. Wilcoxon rank-sum test was used. *P < 0.05, **P < 0.01, ***P < 0.001, *n* = 16.

Synchronization of networks is often reflected by cross-frequency interaction, and the power spectrum could not reflect the phase information. In order to measure the degree of phase couplings at different frequency bands, we computed the filtered wavelet bicoherence (FIWBIC) values with general harmonic wavelet bicoherence [35]. As shown in Figure 4, the FIWBIC values were divided into bands from delta to gamma. A statistical analysis of estimated average of synchronization of FIWBIC values at different bifrequency bands was conducted on the 16 samples from sessions before the incision and compared to that from sessions after the incision. The Wilcoxon rank-sum test at *P* < 0.05 was performed. The boxplots showed that the mean of the synchronization values among the beta, the alpha, and the theta increased, that is, the cross-frequency coupling strength between delta and alpha, and between theta and theta/alpha/beta, between alpha and alpha/beta during the session after the incision was significantly strengthened. Meanwhile, the coupling in the delta band before the incision was higher compared to that after the incision.

### 3.3. EA Treatment-Induced EEG Power Changes in the Incisional Pain Model of Rats

**Figure 5:**
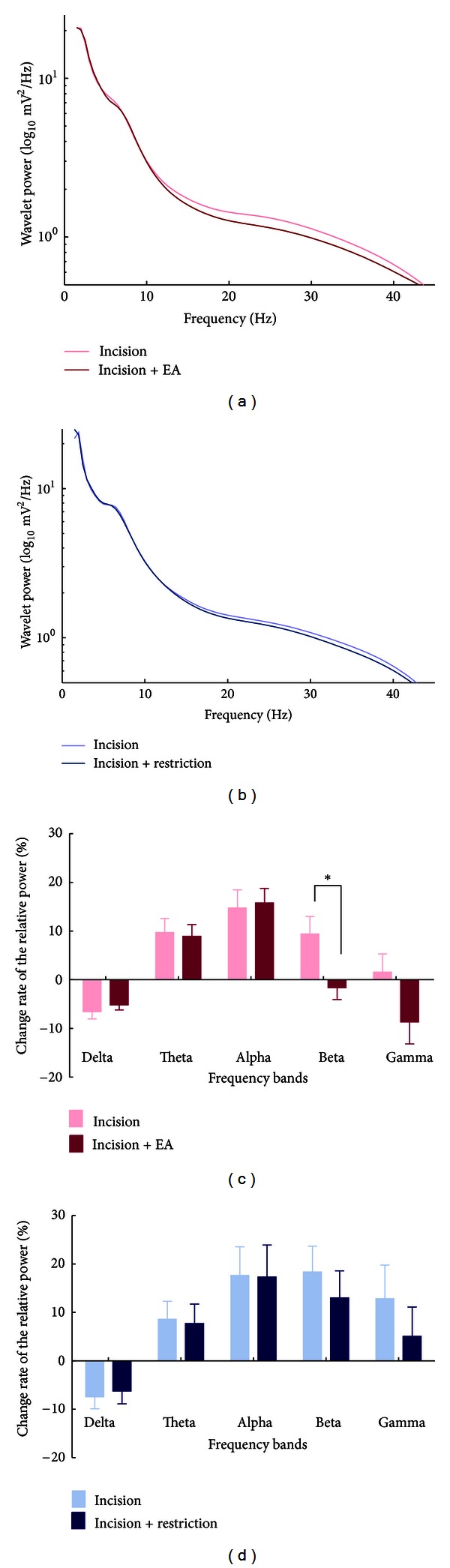
Changes of EEG power spectra after EA treatment in the postincisional pain model of rats. (a) The averaged absolute EEG power after EA treatment (red curve) compared with that before EA treatment (pink curve). The *y*-axis represented power value, and *x*-axis represented frequency bands. (b) The averaged absolute EEG power after restriction (dark blue color) compared with that before restriction (light blue curve). The *y*-axis represents power value, and the *x*-axis represents the frequency bands. (c) Change of the relative power in the EA group. The relative power value in EA group was normalized as the percentage relative to that before the incision. There was a significant decrease at the beta band after EA (red bar) compared with that after the incision (pink bar). (d) Change of the relative power in the restriction group. No significant changes were observed (light blue bar versus dark blue bar). *P < 0.05 (paired *t*-test).

In view of behavior results described above, both the sham EA treatment group and the restriction (without EA) group showed similar pain behaviors, and we chose the restriction group as control. The 16 rats were randomized into two groups: one group received EA application for 30 min from 2 h to 2.5 h after the plantar incision, and the other remained in restriction as control. We compared the power change at different oscillation bands in two groups (Figure 5). Figures 5(a) and 5(b) showed the change rate of averaged absolute power after EA treatment or restriction, separately. Figures 5(c) and 5(d) presented the change rate of relative power during the sessions before and after the EA application relative to the session before the incision in two groups, respectively. Only after EA treatment, the negative change rate relative to the baseline (before the incision) showed significant reduction in high-frequency bands, especially in the beta band. A tendency of reduction was also found in the gamma band. Additionally, the change of power values during the two sessions before EA or restriction was not different between two groups (Figure S2).

**Figure 6:**
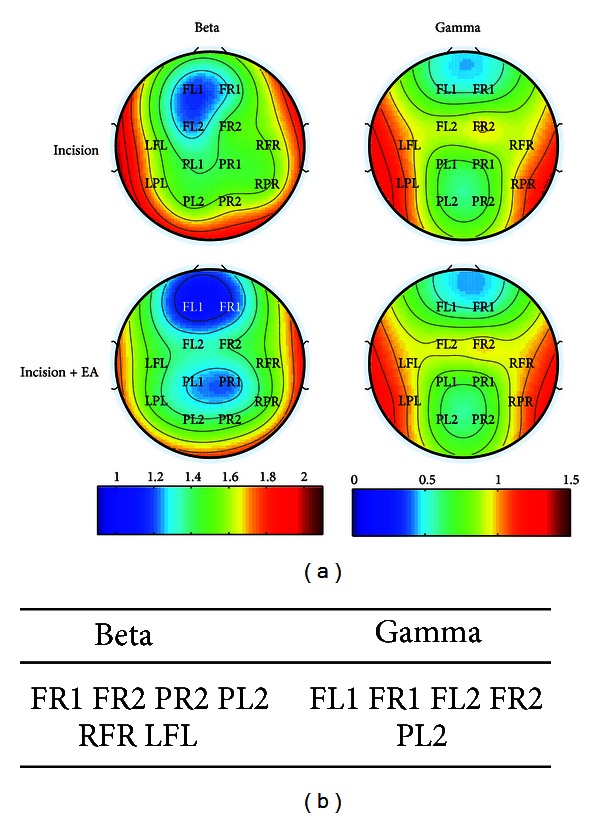
Locations of electrodes with significant EEG power change after EA treatment in incisional pain model of rats. (a) Topographic mapping of EEG power in beta band (left) and gamma band (right) after EA application compared with that before EA application. Averaged EEG power density values were color-coded and plotted at the corresponding position on the planar projection of the epidural surface and interpolated between electrodes (dots). (b) Electrodes with statistically significant difference (*P* < 0.05) of power change in the beta and gamma band.

Furthermore, after EA application, the change at the beta band mainly located over the electrodes of FR1, FR2, PR2, PL2, RFR, and LFL (Figure 6(b)), corresponding to the right fronto-parietal lobe, the left posterior parietal lobe, and the bilateral temporal lobes; meanwhile, the change at the gamma band located in the bilateral frontal lobes and the left posterior parietal lobe according to the location of electrodes (Figure 6(b)).

**Figure 7:**
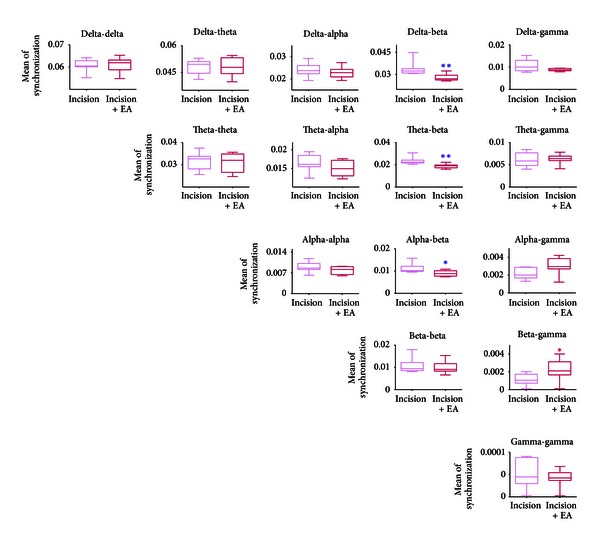
Phase couplings at different frequency bands: statistical analysis of the filtered wavelet bicoherence (FIWBIC) values after EA treatment in the postincisional pain model of rats. Boxplots stand for the phase coupling at local frequency bands, and the *y*-axis represents the mean of synchronization. After EA application, the synchronization value between beta and delta/theta/alpha decreased, that is, synchronization became weaker; whereas the value between beta and gamma increased, that is, synchronization became stronger. Wilcoxon rank-sum test was used. *P < 0.05, **P < 0.01, ***P < 0.001, *n* = 8.

Similarly, we computed FIWBIC values in EA treatment group and the restriction group, respectively. The statistical results of Wilcoxon rank-sum tests at *P* < 0.05 at the different frequency bands in two groups are shown in Figure 7 and Figure S3, respectively. From Figure 7, it is shown that after EA treatment, the strength of bifrequency coupling between beta and delta/theta/alpha attenuated significantly, whereas the coupling of beta-gamma bands became greater. In contrast, no significant difference was found in the strength of phase coupling between bands after restriction compared with that after the incision (Figure S3). 

## 4. Discussion

In the incisional pain model of rats, by analysis of power spectrum and bicoherence of EEG, we found that the ongoing activities at the delta band decreased, while the activities at theta, alpha, and beta bands increased in the plantar-incisional pain rats; EA treatment decreased the power in high-frequency bands, especially at the beta band, and reversed the enhancement of the cross-frequency coupling strength between the beta and low-frequency bands.

### 4.1. Brain EEG Oscillations in Postincisional Pain

With rat EEG, we observed significant changes of the ongoing power spectra in different frequency oscillations ranging from the delta band to the beta band except the gamma band we also noticed the obvious changes of bi-frequency coherence during the postincisional pain. It is well known that pain perception is a multidimensional experience with sensory-discriminative, affective-emotional, and cognitive-evaluative components [3], and different brain areas are involved in different dimensions. Moreover, different cognitive systems are related to neuronal networks of different sizes and distribution, networks of different sizes oscillate at different frequencies, and mutual interactions of cross-frequency oscillations could be well positioned to regulate the multinetwork integration [36]. Thus, it might be conceivable that extensive range of neuronal oscillations participated in pathological pain cortical processing; any oscillation itself is not sufficient for integrating all the distributed information required for pain perception.

It has been accepted that delta-band oscillation is associated with compromised neuronal function [37]; theta-band is linked with emotional arousal [38]; beta-band may relate to the maintenance of the current sensorimotor or cognitive state, and its pathological enhancement may result in an abnormal persistence of the status quo and a deterioration of flexible behavior and cognitive control [39]. Thus, in our results, the reduced delta activity, as well as the increased theta and beta activities, might reflect the cortical overactivation induced by different pain dimensions in the resting state. Alpha oscillation is well known to mainly serve as a top-down controlled inhibitory mechanism [38]. One possible explanation for the increased alpha oscillation is that the evaluative component of pain, represented by paw lifting, was weakened by the activation of the descending pain inhibition network. Meanwhile, no significant change in the gamma oscillation was found during the postincisional pain. Owning that the gamma band is well recognized to be linked with feature binding, working memory, attention, or sensory selection [1], whereas our current study focused on the sustained, spontaneous pain in acute stage, it may be speculated that sensory selection or pain memory was not engaged in the pain processing. More importantly, the findings on alterations of phase coupling strength across distinct oscillations might reflect the synchronization of neuronal networks involved in different pain dimensions and the integration of pain inhibition and facilitation networks. It is also in accordance with the idea of neuronal processing with various simultaneous oscillations [36].

Topography findings showed the change of power in different frequency bands mainly located over bilateral frontal and parietal cortices (Figure 3). These are locations of the primary somatosensory cortex and the anterior cingulate cortex in rat. In this study, we selected the recording session from 1.5 to 2 h after the plantar incision. Given anatomical and physiological connections between right and left hemispheres, it was reasonable that the change of power occurred in bilateral cortices via communication and integration of persistent painful information in the whole brain.

### 4.2. EA Modulation on Incisional Pain-Related Brain Oscillations

In the present study, we investigated the modulation of EA treatment on the incisional pain-related brain oscillations in rats. As a basis, we firstly investigated the analgesic effects of EA treatment in the incisional pain rats. We found that EA treatment on “Zusanli” and “Sanyinjiao” acupoints on bilateral sides in hind paws relieved the mechanical allodynia in rats after the plantar incision. These results, in line with other reports [11–13], suggested that EA was effective in relieving plantar incisional pain and this antinociceptive effect could maintain at least 30 min.

We further explored the neural oscillation mechanism of EA analgesia. From our results, brain oscillations induced by EA application and those in nociceptive processing were not identical; the change of power after EA occurred at the beta band. It provides evidence that neuronal networks participated in EA treatment are different from those in pain perception. The networks oscillate at different frequencies, although brain areas activated by both EA treatment and incisional pain itself have large overlap from imaging studies [14, 15, 40], which is also in line with our results of topographic mapping. 

As we mentioned before, the enhancement of the beta oscillation might be linked to the deterioration of flexible behavior and cognitive control [39]. Accumulating evidence suggests that EA facilitates the descending pain inhibitory pathway by increasing the release of opioid peptides in the central nervous system. Therefore, in our results, the decrease of activities at the beta band is reasonable, because EA treatment facilitates the descending inhibitory system of pain.

Prior studies indicated that inhibitory GABAergic interneurons network played a key role in the modulation of beta and gamma oscillations. Elevated endogenous GABA levels could cause the elevation of beta power [41, 42]. In the EA antinociceptive effects, GABA agonists showed reverse interaction with opioid receptor agonists [43, 44]. In addition, EA could decrease intracerebral GABA content in the cortex on the lesioned side in the rat model of Parkinson's disease [45]. Taken together, it is conceivable that the decreased power of high-frequency oscillations in our results might reflect the inhibition of GABAergic interneurons induced by EA application in pain situation. 

It has been accepted that low-frequency oscillations might be involved in the integration across widely spatially distributed neural assemblies and high-frequency oscillations (beta and gamma bands) distributed over a more limited topographic area. The integration of different local high-frequency oscillations is mediated by the large scale interactions of low-frequency oscillations. By analysis of the cross-frequency couplings, we found that EA treatment reversed the enhancement of the couplings between low-frequency bands and beta band in incisional pain and also strengthened the couplings between beta and gamma band. It may be speculated that EA exerted antinociceptive effect by modulation on power and cross-frequency coupling strength, which disturbed the cortex excitability and the multinetwork integration of nociceptive information in incisional pain.

In conclusion, the present study suggests that broad frequency oscillations ranging from delta-to beta-frequency bands are correlated with the cortical processing of the nociceptive information in the plantar incisional pain rats. EA reverses the increased beta power and the cross-frequency couplings between the beta and low-frequency bands induced by postincisional pain, suggesting that EA could regulate the neuronal networks involved in the central processing and the integration of spontaneous nociceptive information. These results can deepen our understanding in the central neuromodulatory mechanisms of EA analgesia. 

## Supplementary Material

The EEG recording sites were clarified by the schematic drawing of the electrodes' spatial distribution. The possibility of grouping differences causing the EA-induced power change was excluded from unpaired *t*-test statistics between the two groups. No significant changes of cross-frequency couplings after restriction in restriction group confirmed that the changes of cross-frequency couplings strength induced by EA treatment were special.
