# Supplementary material for: Modulation of Brain Electroencephalography Oscillations by Electroacupuncture in a Rat Model of Postincisional Pain
Source: Evid Based Complement Alternat Med. 2013 Apr 28;2013:160357. doi: 10.1155/2013/160357 (PMC3655616; doi:10.1155/2013/160357)
Supplement: Supplementary file 1 — The EEG recording sites were clarified by the schematic drawing of the electrodes' spatial distribution. The possibility of grouping differences causing the EA-induced power change was excluded from unpaired t-test statistics between the two groups. No significant changes of cross-frequency couplings after restriction in restriction group confirmed that the changes of cross-frequency couplings strength induced by EA treatment were special. [file 160357.f1.pdf]

Figure legends

FIGURE S1: Spatial distribution of the electrodes.

FIGURE S2: Comparison of absolute power during the first two sessions between EA and restriction group. (A) Comparison of power values during the session before the incision between two groups. No significant differences were found across different frequency bands. (B) Comparison of power values during the session after the incision between two groups. No difference was presented in any bands. n = 8.

FIGURE S3：Phase couplings at different frequency bands: statistical analysis of the filtered wavelet bicoherence (FIWBIC) values after the restriction in the post-incisional pain model of rats. Boxplots stand for the phase coupling at local frequency bands, and the Y-axis represents the mean of synchronization. After restriction, the synchronization values among all frequency bands did not show any significant change. Wilcoxon rank–sum test was used. n = 8.

FIGURE S1

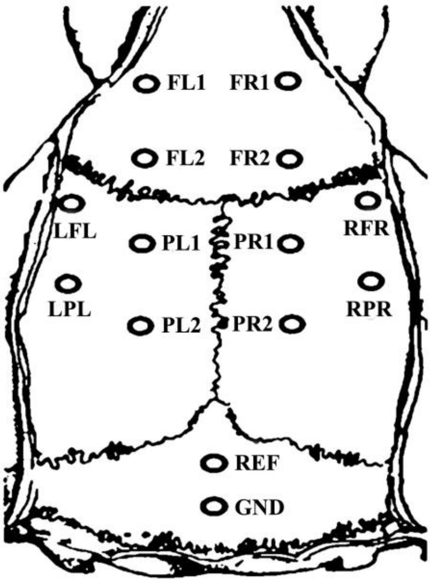

FIGURE S2

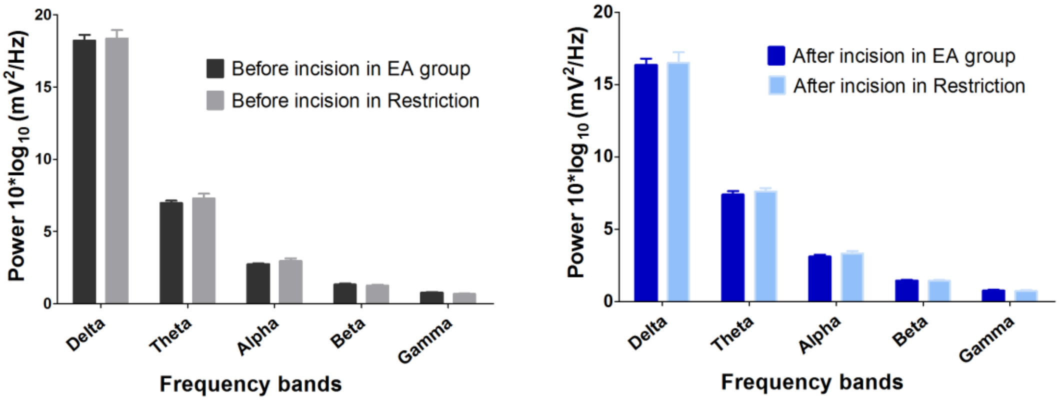
Power 10*log10 (mV2/Hz)
Before incision in EA group
Before incision in Restriction
Delta
Theta
Alpha
Beta
Gamma
Frequency bands
After incision in EA group
After incision in Restriction
Frequency bands

FIGURE S3

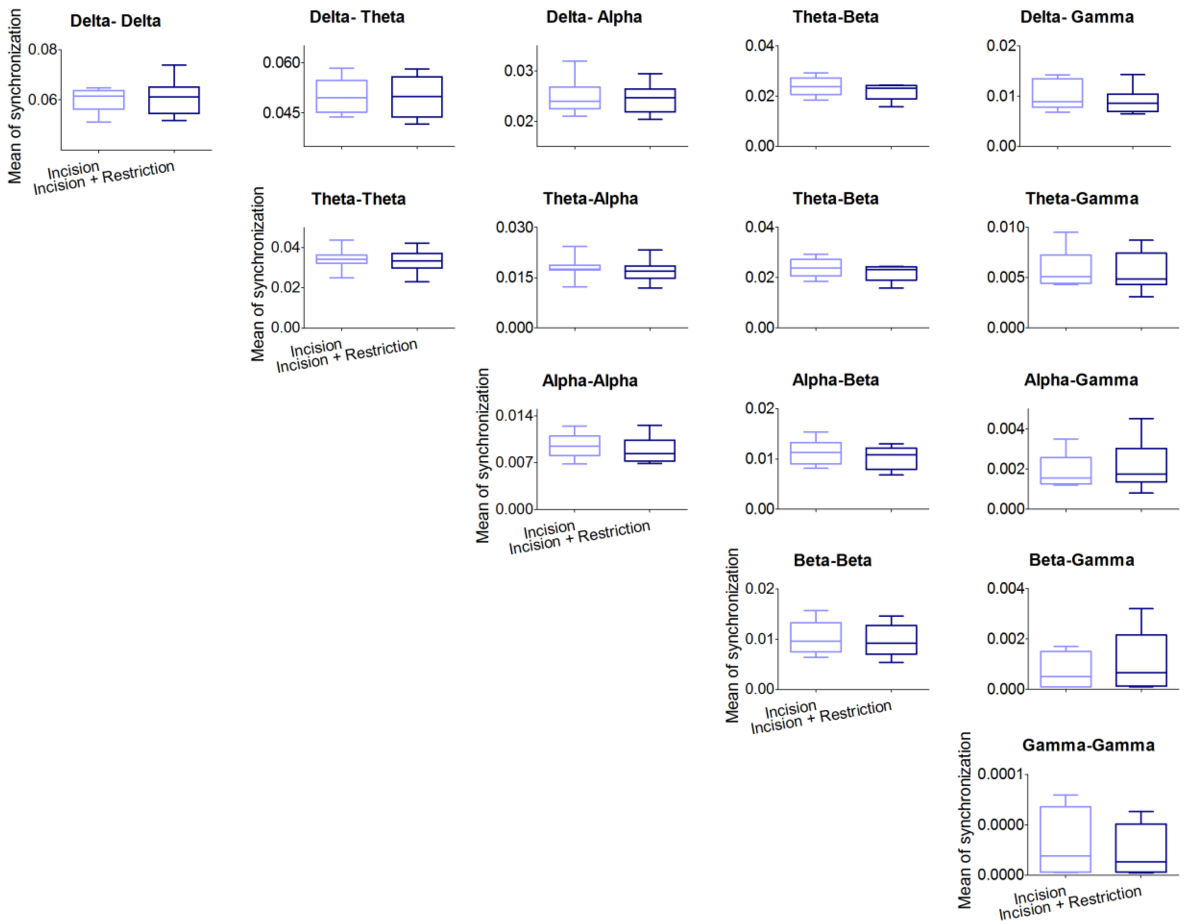
Delta- Delta
Delta- Theta
Delta- Alpha
Theta-Beta
Delta- Gamma
Theta-Theta
Theta-Alpha
Theta-Beta
Theta-Gamma
Alpha-Alpha
Alpha-Beta
Alpha-Gamma
Beta-Beta
Beta-Gamma
Gamma-Gamma
Mean of synchronization
Incision
Incision + Restriction
